# Acute Macular Neuroretinopathy Following COVID-19 mRNA Vaccination

**DOI:** 10.7759/cureus.27502

**Published:** 2022-07-31

**Authors:** Andrew T Rennie, Alexander J DeWeerd, Maria G Martinez, Christine N Kay

**Affiliations:** 1 Clinical Sciences, Florida State University College of Medicine, Tallahassee, USA; 2 Clinical Sciences, Vitreo Retinal Associates, Gainesville, USA; 3 Vitreoretinal Surgery, Vitreo Retinal Associates, Gainesville, USA

**Keywords:** amn, thromboembolic event, vision loss, mrna-based vaccine, retina hemorrhage, macula, moderna vaccine, covid-19, acute macular neuroretinopathy

## Abstract

A 21-year-old female developed bilateral acute-onset paracentral scotomas three days after receiving the second dose of her Moderna COVID-19 vaccination. A clinical diagnosis of acute macular neuroretinopathy (AMN) was confirmed after classic findings were demonstrated on near-infrared reflectance imaging, spectral-domain optical coherence tomography, and colored fundus photography. The patient presented with visual acuity of 20/100-1 OD and 20/20 OS. After treatment with brimonidine and difluprednate, at a two-week follow-up, her visual acuity was 20/100-2 OD and 20/25-2 OS. There have been reported cases of AMN following flu-like illnesses as well as after receiving vaccines. However, this is the first report of AMN following vaccination with a Moderna COVID-19 vaccine.

## Introduction

In the spring of 2020, the SARS-CoV-2 virus launched the world into a pandemic. Multiple vaccines were developed at an unprecedented rate due to a concerted worldwide effort. Pfizer-BioNTech, Moderna, Oxford-AstraZeneca, and Johnson & Johnson (J&J) were some of the companies that developed a vaccine against COVID-19. Pfizer-BioNTech and Moderna produced mRNA-based vaccines, whereas the vaccines produced by Oxford-AstraZeneca and J&J are adenovirus-based vaccines. In March 2021, there was a halt on the distribution of the AstraZeneca vaccine in several European countries due to a concern that the vaccine may lead to increased thromboembolic events [1]. There have also been cases of thrombosis with thrombocytopenia syndrome (TTS) following vaccination with the J&J vaccine [2]. A recent study concluded that the risk of a thromboembolic event after vaccination with Moderna is similar to the risk associated with oral contraceptive use [3].

Acute macular neuroretinopathy (AMN) was first described by Bos and Deutman in 1975 [4]. AMN is a rare retinal disorder that commonly affects young women and typically presents with sudden onset of paracentral scotomas. While the etiology is not entirely clear, it is thought that AMN may be due to an ischemic event disrupting the deep retinal capillary plexus [5]. A 2016 major review of AMN studied 156 eyes in 101 different cases and identified associations of nonspecific flu-like illness or fever (47.5%) and the use of contraceptives (35.6%) in those cases. Most of the patients included in the review were female (84.2%) with a mean age of 29.5 years [6]. Previous reports have indicated the development of AMN following COVID-19 vaccination, each of these limited cases has occurred following vaccination with the Oxford-AstraZeneca vaccine [7-10]. There has been an additional report of AMN following COVID-19 vaccination with the Pfizer vaccine [11]. We report a case of AMN in a young woman three days after receiving the second dose of the Moderna COVID-19 vaccine.

## Case presentation

A 21-year-old female was referred for an ophthalmologic examination due to the sudden onset of bilateral scotomas three days after receiving the second dose of the Moderna COVID-19 vaccine. The patient denied any flu-like symptoms or any febrile illness prior to or following the administration of the vaccine. Her only medication was an oral contraceptive (combined drospirenone and ethinylestradiol).

**Figure 1:**
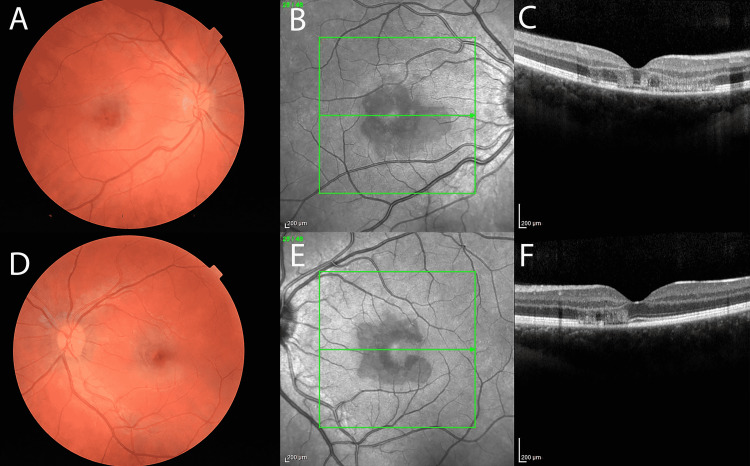
Ophthalmologic imaging upon initial presentation Color fundoscopic images demonstrating perifoveal intraretinal hemorrhages OD with reddish-brown perifoveal petalloid lesions OU (A, D). Near-infrared reflective imaging displays a hyporeflective, wedge-shaped lesion extending nasally from the fovea OD (B) and a horseshoe-shaped lesion nasally to the fovea OS (E). Spectral-domain OCT of OD (C) and OS (F) demonstrate bilateral EZ disruption with hyperreflectivity in the ONL and OPL. OCT: Optical coherence tomography; EZ: Ellipsoid zone; ONL: Outer nuclear layer; OPL: Outer plexiform layer.

On ocular exam, the patient’s visual acuity (VA) was 20/100-1, PH 20/70+1 OD, and 20/20 OS. The intraocular pressure was 19 OD and 19 OS. The anterior chamber was unremarkable OU. Dilated examination of the right eye showed a single anterior vitreous cell, a perifoveal intraretinal hemorrhage, and reddish-brown perifoveal lesions with normal caliber retinal vessels. Dilated examination of the left eye showed a clear vitreous with similar reddish-brown petalloid perifoveal lesions, with normal caliber retinal vessels. Near-infrared reflectance (NIR) imaging in the right eye revealed a well-demarcated, hyporeflective, wedge-shaped macular lesion involving the fovea and extending nasally (Figure 1, Panel B). NIR imaging in the left eye revealed a well-demarcated, hyporeflective, horseshoe-shaped lesion surrounding the fovea and extending nasally (Figure 1, Panel E). Spectral-domain optical coherence tomography (SD-OCT), using the SPECTRALIS system (Heidelberg Engineering, Germany), demonstrated ellipsoid zone (EZ) disruption with intraretinal hemorrhages and hyperreflectivity in the outer nuclear layer (ONL) and outer plexiform layer (OPL) bilaterally (Figure 1, Panels C and F). Fluorescein angiography demonstrated normal AV (arteriovenous) transit with no leakage in either eye. Given the acute development of these characteristic findings along with her clinical history, the patient was diagnosed with AMN. Although no known treatment for AMN exists, options for a trial of a short course of steroids were discussed, and the treatment was initiated with difluprednate 0.05% four times a day OU and a single 20 mg dose of oral prednisone. Options for oral steroids were discussed with the patient and family, but it was decided not to pursue any longer duration of oral prednisone, given the lack of definite efficacy in AMN and desire not to inhibit her immune response to the COVID vaccination.

**Figure 2:**
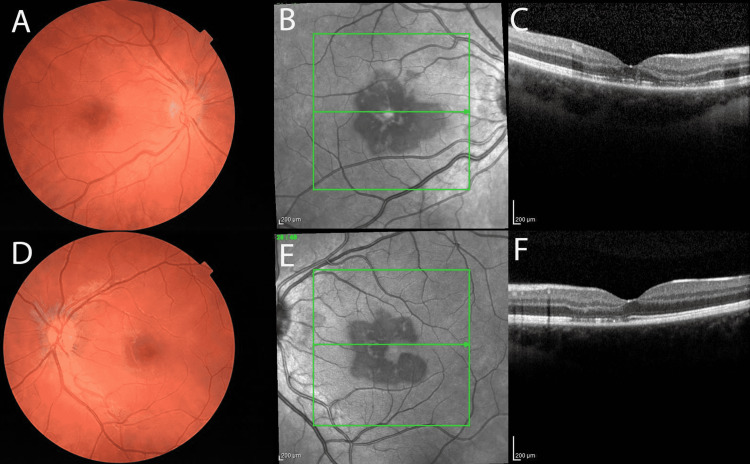
One-week follow-up imaging Color fundoscopic images demonstrating slightly improved perifoveal intraretinal hemorrhages (A, D). Near-infrared reflective imaging displays subtle darkening of the hyporeflective changes from the initial visit (B, E). Spectral-domain OCT demonstrates persistent EZ disruption with some interval resolution of the ONL hyperreflectivity (C, F). OCT: Optical coherence tomography; EZ: Ellipsoid zone; ONL: Outer nuclear layer.

At one-week follow-up, the patient reported no improvement OU. The patient had been using the difluprednate 0.05% four times daily as prescribed. On exam, her VA was 20/200+1, PH 20/50-2 OD, and 20/20-2 OS. The patient’s intraocular pressures were 19 OD and 18 OS. Dilated examination revealed a resolved parafoveal intraretinal hemorrhage with persistent red-brown perifoveal lesions and underlying linear pigmentary disturbances OD. Findings were notable for perifoveal persistent lesions with interval deepening of pigmentary linear perifoveal changes. Color fundus imaging demonstrated persistent perifoveal reddish-brown lesions OU (Figure 2, Panels A and C). NIR continued to reveal interval darkening of hyporeflective petalloid perifoveal lesions OU, and SD-OCT imaging demonstrated persistent EZ disruption with some interval resolution of the ONL hyperreflectivity OU (Figure 2). Microperimetry was performed (Macular Integrity Assessment/MAIA microperimeter) and revealed dense paracentral scotomas OU with mean sensitivities of 22.6 dB OD and 23.6 dB OS, further supporting previous findings. Fundus autofluorescence showed trace perifoveal linear hyporeflective changes (Figure 4, Panels A and B). The patient was continued on difluprednate four times daily OU and recommended to consult with her obstetrician (OB) regarding stopping her contraceptive medication.

**Figure 3:**
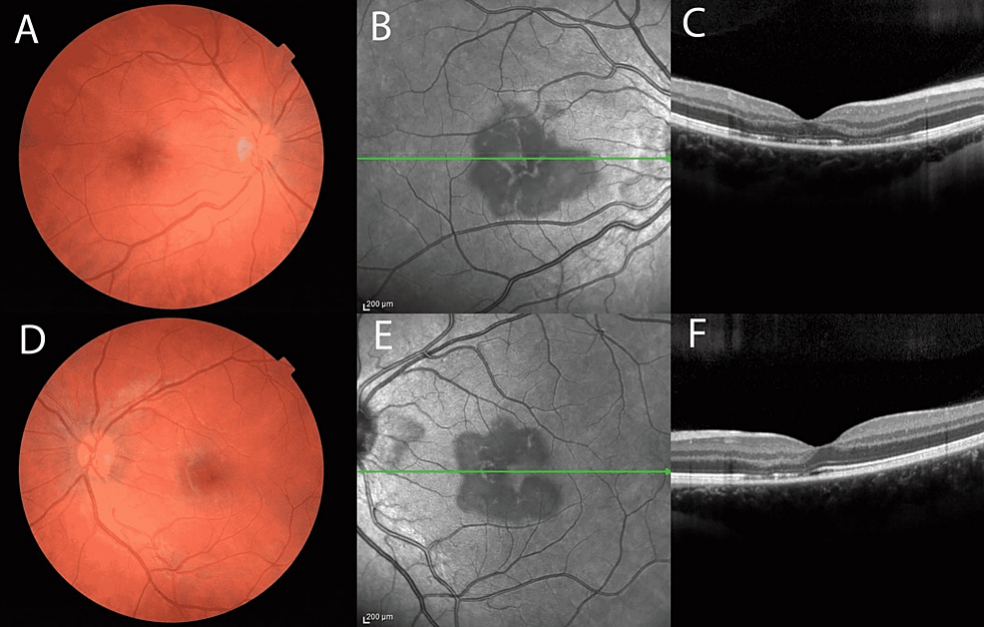
Two-week follow-up imaging Color fundoscopic images demonstrating resolved parafoveal intraretinal hemorrhages with persistent reddish-brown perifoveal lesions OU (A, D). Near-infrared reflective imaging reveals persistent petalloid perifoveal changes OU (B, E). Spectral-domain OCT demonstrates fading opacities, persistent EZ changes, and interval thinning of the ONL (C, F). OCT: Optical coherence tomography; EZ: Ellipsoid zone; ONL: Outer nuclear layer.

**Figure 4:**
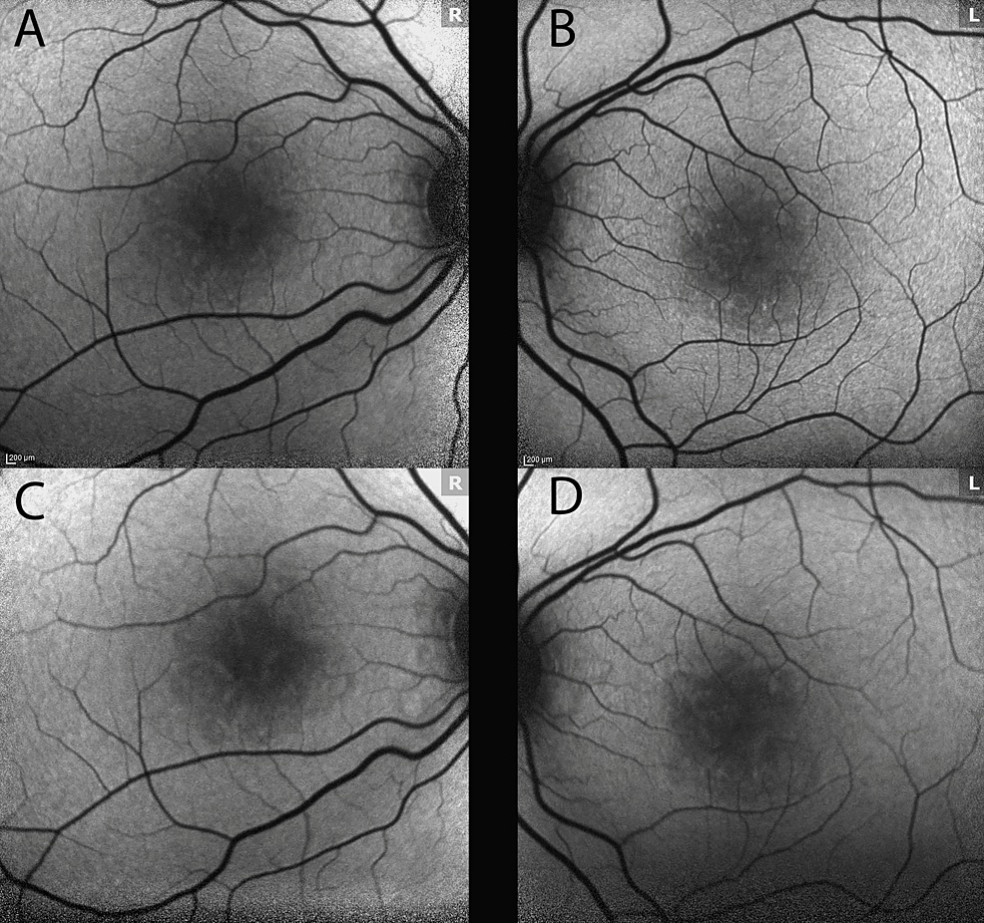
Fundus autofluorescence imaging Fundus autofluorescence imaging performed during one-week (A, B) and two-week (C, D) follow-up visits show faint linear perifoveal irregularities OU more notable at week 2.

At a two-week follow-up, the patient reported no improvement in VA OU. The patient had been continuing to use the difluprednate as prescribed. After discussing with her OB, she opted to stop her oral contraceptive. On exam, her VA was Dsc 20/100-2, PH 20/60+2 OD, and 20/25-2 OS. Her intraocular pressures were elevated at 23 OU, so difluprednate 0.05% was dropped to TID OU and brimonidine 0.1% TID OU was added. Color fundus imaging revealed persistent but fading perifoveal lesions OU (Figure 3, Panels A and C). NIR and SD-OCT were largely unchanged from the previous visit with persistent EZ disruption with fading opacities and interval thinning of the ONL OU (Figure 3). Fundus autofluorescence showed slightly more prominent trace perifoveal linear hyporeflective changes OU (Figure 4, Panels C and D).

## Discussion

This is a rare case of AMN following COVID-19 vaccination with a Moderna vaccine. There have been previous reports of AMN following COVID-19 vaccination; however, these all have occurred following vaccination with the Oxford-AstraZeneca Vaxzevria and a single case following Pfizer-BioNTech vaccination [7-11]. AMN has also been reported following the recovery of confirmed COVID-19 infection [12]. Although an exact pathophysiology of AMN is not yet understood, these studies demonstrate a recurring trend in associated risk factors and disease onset: young females in their 20s who take oral contraceptives that develop paracentral scotomas within one week of a nonspecific flu-like illness.

The current literature on AMN postulates that a vascular compromise within the deep retinal capillary plexus is responsible for the vision impairment seen in patients who develop this condition [5]. In a review on retinal vasculitis, El-Asrar et al. described how systemic inflammation in autoimmune diseases may also impact the vasculature of the eye and cause small-vessel occlusion by microthrombosis, resulting in ischemic retinopathy [13]. As the blood supply to these affected regions is lost, the remaining capillaries deteriorate, and leakage of their contents can be seen as retinal hemorrhages. In AMN, characteristic parafoveal hemorrhages are seen on fundoscopic examination, and SD-OCT findings show hyperreflectivity in the ONL and OPL and EZ disruption, suggestive of damage at the level of photoreceptors [14].

While there is no way to explicitly prove causation between the Moderna vaccine and this patient's development of AMN, there is a growing body of literature to support the association. Further, studies have reviewed and helped to demonstrate the role COVID-19 infection has in the development of microvascular thrombosis and endothelial cell dysfunction [15]. These studies explain how the expression of the angiotensin-converting enzyme-2 (ACE-2) receptor, which is the well-known cell-surface target for COVID-19 viral particles, throughout the brain endothelial and smooth muscle cells may increase the susceptibility to neurologic involvement and microvascular angiopathy following COVID-19 infection and in patients with a hyperimmune response.

The role of vaccines remains extremely important, especially for those with immunocompromised states. In the formulation of vaccines, there are often adjuvants added that assist in boosting vaccine efficacy. Typically, this is without consequence, and the intended goal is achieved. However, in a small subset of patients, there is an activation of an autoimmune or inflammatory response as a result. Within the COVID-19 mRNA vaccines are similar adjuvants that can stimulate an exaggerated immune response following vaccination in individuals, particularly those with autoimmune diseases [16]. In this population, dysregulation between the innate and adaptive immune systems has been shown to be linked with the immune-mediated disease development found in some individuals following exposure to an adjuvant. While it cannot be stated with certainty that the patient in our case suffered AMN because of adjuvant exposure, the temporal relationship between her mRNA vaccination and clinical symptoms along with a co-existing hypercoagulable state may represent a rare, nonspecific association connecting these phenomena.

## Conclusions

The 21-year-old female patient in our case demonstrated classic fundoscopic, SD-OCT, and NIR findings of AMN three days after the second dose of her Moderna COVID-19 vaccination. The use of vaccination is safe and effective, having drastically reduced the risk of morbidity and mortality associated with COVID-related disease. However, there remains a possibility for rare adverse events to occur in a very small subset of patients. From our review of the literature, this is the first report of the AMN post-Moderna COVID vaccine, making this an extremely rare clinical entity.
